# Adolescent ACL Reconstruction Using Quadriceps or Hamstring Tendon Autografts: A Comparative Study of Muscle Strength and Patient-Reported Outcomes

**DOI:** 10.3390/jcm14113842

**Published:** 2025-05-29

**Authors:** Ritauras Rakauskas, Laimonas Šiupšinskas, Vytautas Streckis, Laurynas Galinskas, Rokas Jurkonis, Jūratė Tomkevičiūtė, Dalius Malcius, Emilis Čekanauskas

**Affiliations:** 1 Department of Paediatric Surgery, Lithuanian University of Health Sciences, LT-44307 Kaunas, Lithuania; dalius.malcius@lsmu.lt (D.M.); emilis.cekanauskas@lsmu.lt (E.Č.); 2 Department of Sports Medicine, Lithuanian University of Health Sciences, LT-44307 Kaunas, Lithuania; laimonas.siupsinskas@lsmu.lt; 3 Institute of Sports Science and Innovations, Lithuanian Sports University, LT-44221 Kaunas, Lithuania; vytautas.streckis@lsu.lt; 4 Department of Trainings Systems, Lithuanian Sports University, LT-44221 Kaunas, Lithuania; 5 Department of Orthopaedics and Traumatology, Lithuanian University of Health Sciences, LT-44307 Kaunas, Lithuania; laurynas.galinskas@stud.lsmu.lt (L.G.); rokas.jurkonis@lsmu.lt (R.J.); 6 Department of Physics, Mathematics and Biophysics, Lithuanian University of Health Sciences, LT-44307 Kaunas, Lithuania; jurate.tomkeviciute@lsmu.lt

**Keywords:** adolescent, anterior cruciate ligament reconstruction, autografts, hamstring tendons, muscle strength, patient satisfaction, quadriceps muscle, treatment outcome

## Abstract

**Background and Objectives:** A prominent area of research in adolescent ACL reconstruction (ACLR) involves graft selection, with particular interest in the quadriceps tendon (QT) as an alternative to hamstring tendon (HT) autografts, although comparative studies on muscle strength outcomes and patient satisfaction in adolescents remain limited. This study aims to evaluate the influence of QT and HT autografts on muscle strength and satisfaction outcomes post-ACLR in adolescents. **Methods:** This prospective study was conducted at the Lithuanian University of Health Sciences, enrolling 68 patients aged 12–17. ACLRs were performed using QT or HT autografts. Muscle strength was assessed using an isokinetic dynamometer (Biodex), while patient satisfaction was evaluated through IKDC, Lysholm, and ACL-RSI surveys preoperatively and 12 months postoperatively. **Results:** 54 patients (24 QT and 30 HT) completed the study. The data are non-parametric and are presented as median (IQR). Isokinetic measurements indicated that QT harvesting led to significantly lower extension strength compared to HT 6 months (*p* = 0.019) and 12 months post-op (*p* < 0.001) but showed better H/Q ratios 6 months (*p* = 0.019) and 12 months post-op (*p* < 0.001). There was no significant difference between the QT and HT groups in ACL-RSI and Lysholm scores. IKDC scores were significantly higher in the HT group (*p* = 0.009). **Conclusions:** QT autografts provide favorable H/Q ratios, yet they exhibit weaker extension strength and lower satisfaction outcomes compared to HT. These results suggest a need for individualized rehabilitation protocols and further research to optimize ACLR graft selection for adolescents.

## 1. Introduction

A significant increase in youth sports participation, year-round training, competitions, and early sport specialization has contributed to a greater incidence of anterior cruciate ligament (ACL) tears in skeletally immature athletes [1]. Graft selection for anterior cruciate ligament reconstruction (ACLR) remains one of the main areas of research in today’s orthopedics [2]. Over the past 5–6 years, the number of studies investigating the quadriceps tendon (QT) as an excellent alternative to the hamstring tendon (HT) in ACLR has increased dramatically and continues to grow [2,3,4]. However, despite the growing interest, we still lack studies that compare muscle strength outcomes and patient satisfaction in adolescents between those grafts [1].

Witnessing the growth of anterior cruciate ligament (ACL) rupture and re-rupture rates in adolescent patients, it is crucial to make ACLR as stable as possible [5,6]. QT autograft has decent anatomical and biomechanical characteristics [2]. From the previous studies, we can see that it has superior cross-sectional area, stiffness, and ultimate load to failure [7]. Histologically, it has more tenocytes and apparently matures faster than HT, which may have an influence on the lower future re-rupture possibility and make QT a viable graft choice over HT [8,9,10]. Although hamstrings have been utilized as a reliable graft for many years, they are important for knee kinematics and should be spared if possible [2,11]. They act as an ally to the ACL, protect the shin from anterior movement, and provide the knee with valgus/varus and rotational stability [11].

Most existing literature centers on adult cohorts, with limited pediatric or adolescent-specific data. This study is among the first to provide a head-to-head analysis of all-soft-tissue QT versus HT autografts in adolescents undergoing ACLR.

The novelty of this research lies in its integration of quantitative strength assessments with subjective measures of recovery (IKDC and ACL-RSI scores), offering a comprehensive evaluation of muscle strength and psychological recovery.

The purpose of this study is to evaluate the influence of different tendon grafting techniques on patients’ muscle strength and satisfaction outcomes after ACLR. We investigated only all-soft-tissue QT and HT autografts. We are interested in what impact it has on knee extensor and flexor mechanisms, the hamstring-to-quadriceps (H/Q) ratio, and possible re-injury of the operated knee. The hypothesis of this study is that sparing the hamstrings and harvesting QT instead leads to better thigh muscle strength outcomes and patient postoperative satisfaction while reducing the possibility of re-injury.

## 2. Materials and Methods

### 2.1. Participants and Surgical Technique

This prospective comparative study was performed at the Lithuanian University of Health Sciences (LUHS) Kaunas Clinics Department of Pediatric Orthopedics with a total of 68 patients (37 males and 31 females) from 12 to 17 years old. The study was carried out between September of 2021 and March of 2024. The permission to conduct this study was acquired by the Kaunas Regional Biomedical Research Ethics Committee (protocol no: BE-2-103). Before inclusion in this study, all patients or their parents were required to sign informed consent forms.

All patients with an ACL rupture with or without meniscal rupture, as confirmed by MRI, were included in this study. Patients with other knee injuries were excluded from the study.

All clinical cases were primary ACL reconstructions, and patients had no history of previous injuries of the knee.

All ACLRs were performed by a single orthopedic surgeon using a standard trans-epiphyseal drilling technique. HT were harvested through a 3–4 cm anteromedial incision over the pes anserinus. Both the semitendinosus and gracilis tendons were harvested, and 8-strand 7 cm length from 9 to 11.5 mm in diameter grafts were obtained. QT were harvested through a 3–4 cm incision over the distal end of the thigh using a 10 mm wide blade. Grafts were 7 cm in length and from 9 to 11 mm in diameter. For the fixation of the grafts in tunnels, cortical suspensory devices TightRope RT and ABS TightRope, Arthrex (Naples, FL, USA) were used. To make sure that implants were in an adequate position, post-operative X-rays were obtained.

Post-operatively, all patients were immobilized with hinged knee braces and started the first stage of rehabilitation. If the operation was isolated ACLR or ACLR with meniscal resection, initial weight bearing was allowed. Only patients with a repaired meniscus could not bear weight on the operated leg for 4 weeks. In all of our cases, the second stage of rehabilitation started after 6 weeks. Testing with an electromechanical dynamometer Biodex (Shirley, NY, USA) was started only after 6 months, when it was estimated to be safe to perform.

### 2.2. Muscle Strength Evaluation (Isokinetic Measurement) by Biodex

Testing of the thigh flexor and extensor strengths was performed in collaboration with Lithuanian Sports University at their scientific laboratory. All the participants were informed about the process of the test. Participants were asked not to exercise for 24 h and not to eat for at least 2 h before testing. Anthropometric measurements were collected from all participants. The Strength test began with a 10 min warm-up on a stationary exercise bike, Monark (Varberg, Sweden), at an average of 60 to 70 revolutions per minute, intensity of 70 W, and pulse of 110 to 130 beats per minute. The room temperature while testing was 23 °C and 77% humidity. After the warm-up, the participant had to rest for 5 min. During the resting period, the participant was seated on an isokinetic dynamometer Biodex Medical System 4 (Shirley, NY, USA) and their chest, waist, and thigh were secured with fixation straps. The lower leg was attached to the lever arm of the dynamometer at the level proximal to the malleoli. The axis of rotation of the dynamometer lever arm was visually aligned with the axis of the femur. The range of motion of the tested leg was measured, and the gravitational force was calibrated by weighing the leg–foot segment of the participant at 60 ± 5 degrees of flexion [12]. To familiarize themselves with the testing, each participant went through four trials using 25, 50, 75, and 100 percent of their capable force at the set range of motion. During the test, the participant performed three maximal trials at a velocity of 60° per second. Participants were verbally encouraged to provide maximal effort throughout. A rest period of 60 s between the trials was given. The uninvolved leg was tested first, and the involved leg was tested afterwards. The physiological H/Q ratio was considered as 60% [13,14,15]. Testing was conducted at 6 and 12 months after the surgery.

### 2.3. Satisfaction Evaluation

Patient satisfaction with the outcomes after ACLR was assessed before surgery and 12 months after surgery at the last Biodex testing. All of the participants had to fill out the IKDC Knee score, Lysholm score, and ACL-RSI (ACL return to sports after injury) surveys.

### 2.4. Statistical Analysis

An a priori power analysis was performed using IBM SPSS Statistics (version 30) to estimate the sample size required to detect a minimal clinically important 10% difference in the H/Q ratio between the HT and QT groups. We used data from 15 patients in our pilot study. The standard deviation in the HT group was 10.57, and in the QT group, it was 12.48. The power analysis showed a minimum sample size of 23 in each of the HT and QT groups, with chosen significance level α = 0.05 and power of two-sided statistical test 1 − β = 0.8.

Data analysis was carried out using IBM SPSS Statistics version 30. Quantitative variables are summarized with descriptive statistics, including the median and interquartile range (IQR). Categorical (qualitative) data are reported as frequencies and percentages. Due to the small sample sizes and lack of normal distribution (assessed with the Shapiro–Wilk test), comparisons between two independent groups were made using the non-parametric Mann–Whitney U test. Frequencies of categorical variables between groups were compared using the Chi-square test for homogeneity. For comparisons between related (paired) samples, the Wilcoxon signed-rank test was applied. The difference between the compared samples was considered statistically significant if the *p*-value was less than 0.05.

## 3. Results

### 3.1. Sample Characteristics

**Figure 1:**
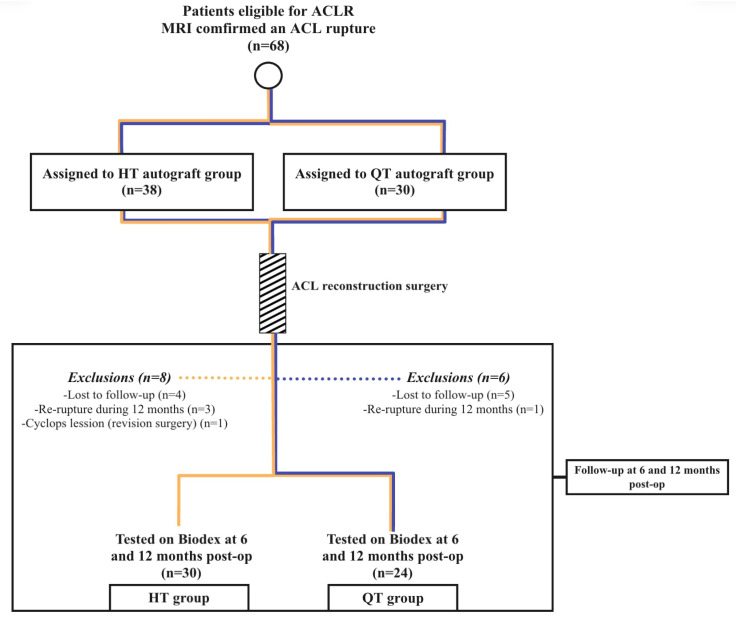
Flow chart of patient follow-up during 12 months of study.

A total of 68 patients participated in this study. Unfortunately, not all participants were able to be followed up with for all 12 months, and 14 patients were lost due to various reasons. The flow chart that describes patient involvement in the study is presented in Figure 1.

Despite various inconveniences, 54 participants (24 in the QT and 30 in the HT group) from 13 to 17 years of age participated in all stages of the study. The closed-envelope method was used to assign patients to the QT or HT group. A total of 49 (90.7%) of the remaining patients in our study sustained ACL injuries while playing some kind of sport. Sports activities varied as follows: 11 (20.37%) played some unspecific sport games, 10 (18.52%)—basketball, 9 (16.67%)—soccer, 7 (12.96%)—handball, 4 (7.41%)—volleyball, 3 (5.56%)—martial arts, 2 (3.7%)—rugby, 2 (3.7%)—skiing, and 1 (1.85%)—athletics. The remaining 5 (9.26%) patients were injured in some form of accident: 3 (5.56%)—road accident, 2 (3.7%)—electric scooter accident, and 1 (1.85%)—fall on the stairs. They completed both Biodex tests and satisfaction evaluation questionnaires. Sample characteristics are presented in Table 1.

### 3.2. Muscle Strength Outcomes Between the Groups

To investigate the thigh muscle strength outcomes between different autograft groups of the kinematics of the knee joint, it was important to find out how different autografts affected the strength of extensor, flexor mechanisms (peak torque to body weight ratio), and H/Q ratio between the graft groups. These results are presented in Table 2. As is shown in this table, the results of comparing the two groups were somewhat different than we expected. It seems that the harvesting of hamstring tendons did not have a significant effect on the reduction in flexion strength, as the flexion in the HT group was not statistically significantly different from the QT group, where the hamstrings were preserved. While comparing the extension strength between the groups, the results were probably logical and could be expected. Quadriceps tendon harvesting affected extension strength, and, in the QT group, the results were significantly lower.

The difference in the change in the extension force of the operated leg was not statistically significant when comparing the QT and HT groups (Mann–Whitney test Z = −1.413, *p* = 0.158).

The change in flexion force in the operated leg was not statistically significant when comparing the QT and HT groups (Mann—Whitney test Z = −0.784, *p* = 0.433).

The results of the ratio between agonist and antagonist muscle groups reveal two key aspects. The H/Q ratio in the QT group is, on average, much closer to our target of 60 percent, which theoretically could lead to lower re-rupture rates in the QT group. However, according to the large deviations between the minimum and maximum, it seems that the favorable H/Q ratio could be the result of the high morbidity of the quadriceps muscle when it was somewhat impaired after the tendon harvest, and the hamstrings seem much stronger than they are in the test.

The difference in the change in the H/Q ratio of the operated leg was not statistically significant when comparing the QT and HT groups (Mann–Whitney test Z = −0.314, *p* = 0.754).

Another notable finding is that a 12-month period was insufficient for the two groups to reach comparable outcomes. Flexion, extension, and their ratio results remained similar between groups at 6 and 12 months.

### 3.3. Muscle Strength Outcomes Comparing Involved and Uninvolved Legs Between the Groups

For the second stage of the study, we compared the results only in the same autograft group between healthy and operated legs. These results are presented in Table 3.

The results in the QT group are similar to what we expected and thus confirm our theory of the quadriceps tendons’ morbidity influence on the better H/Q ratio in the QT group. At both 6 and 12 months, the quadriceps remained weaker in the operated leg, and because of that, the H/Q ratio in the operated leg was much better and closer to the target of 60 percent. We also think that it is possible to conclude that the preservation of the hamstrings had a positive effect on the posterior groups of muscles, because after 6 months, the flexor strength between both legs did not differ.

The change in the flexion force was not statistically significant when comparing the operated and uninvolved leg in either the HT or QT group (in the HT group, Wilcoxon test Z = −0.154, *p* = 0.877; in the QT group, Wilcoxon test Z = −1.202, *p* = 0.229).

For the QT group, the change in the extension force was not statistically significant when comparing the operated and uninvolved leg (Wilcoxon test Z = −0.429, *p* = 0.668).

Some interesting conclusions from the results in the HT group can be drawn, too. Even when HT was chosen as an autograft, the quadriceps muscle was statistically significantly weaker in the operated leg after 6 and 12 months. When evaluating the flexor strength in the HT group, the results were as expected. Flexion strength in the operated leg was weaker after 6 and 12 months due to the harvested hamstring tendons. When evaluating the H/Q ratio, the results are radically different in the QT group. Flexor strength in the operated and healthy leg did not differ after 6 months. Interestingly, the H/Q ratio in the uninvolved leg showed a statistically significant decline at 12 months.

For the HT group, the change in the extension force was statistically significant when comparing the operated and uninvolved leg (Wilcoxon test Z = −2.934, *p* = 0.003). The extension force of the operated leg changed more than that of the uninvolved leg.

The change in the H/Q ratio was statistically significant when comparing the operated and uninvolved legs in both the HT and QT groups (in the HT group, Wilcoxon test Z = −4.108, *p* < 0.001; in the QT group, Wilcoxon test Z = −2.232, *p* = 0.026). The H/Q ratio of the operated leg changed more than in the uninvolved leg.

### 3.4. Satisfaction Outcomes Between the Groups

In the third stage, we evaluated patient satisfaction between both autograft groups 12 months after the surgery. Although the results in all three questionnaires were lower in the QT group, they were only statistically significant in the IKDC score. This could be related to the morbidity effect of the quadriceps muscle because of the harvested part of the tendon. The results are presented in Table 4.

## 4. Discussion

There is still a lack of evidence surrounding the comparisons of different autografts on muscle strength outcomes, failure rates, and patient satisfaction for pediatric ACLR [1]. Fischer et al. (2017), in their study, compared isokinetic quadriceps and hamstring muscle strength in patients following ACLR who received either HT autografts or QT autografts, with no significant differences between groups and no effect of age [16].

It is expected, depending on the site of harvesting, that the peak knee flexion torque would be lower in the HT group, and the peak knee extension torque would be lower in the QT group, postoperatively. According to our data, harvesting the quadriceps tendon seems to have a negative impact on muscle strength outcomes and patient satisfaction during the 12-month follow-up after ACLR. While the QT graft H/Q ratio after ACLR is almost ideal and theoretically reduces the re-rupture rate, it seems that this is a result of iatrogenic injury to the quadriceps muscle and does not reveal the real strength of the hamstrings. It also may result in lower patient satisfaction after ACLR.

Moreover, a higher H/Q ratio in the QT group suggests better muscle balance around the knee joint and may be a protective factor in recurrent ACL injuries [16]. Martin-Alguacil et al. (2018) conversely suggest that the flexion strength deficits in the HT group, and thus the H/Q imbalance measured in this group, increase the risk of graft rupture in patients similar to those included in this randomized control trial (young soccer players) [17].

Other authors, in similar studies, have published results that still promote QT as a viable graft for ACLR. J. D. Hughes et al. (2019), in their similar short-term study where they evaluated muscle strength outcomes using Biodex after ACLR with QT, HT, and patellar tendon (BPTB), obtained better results [18]. Although at 5 to 8 months QT group had weaker quadriceps muscle, at 9 to 15 months, there was no difference between the groups. They noted that more emphasized rehabilitation programs may be key to reducing residual quadriceps weakness. De Petrillo et al. (2022) in their study concluded that the QT, HT, and BPTB autografts lead to specific short-term muscle strength deficits post-ACLR in pediatric patients that should be considered concomitantly with patient-specific factors, and adequate rehabilitation protocols can help minimize those muscle strength deficits [1]. J. Kay et al. (2023), in their retrospective study, similarly found that adolescents who underwent ACLR with QT had greater quadriceps strength deficits, while the ones with HT grafts had lower flexion strength [19]. All of this led to a better H/Q ratio in the QT group and had no influence on hopping. C. Horteur et al. (2021) reported even better results. They reported that QT harvesting is a pejorative factor for functional outcomes and does not yield significant quadriceps weakness [20].

We hypothesize that one of the primary factors in why patients in our study had such a high residual quadriceps weakness is that all patients had the same rehabilitation program, no matter what autograft was used. I. Setuain et al. (2017), in their article, evaluated objective-care- and usual-care-based programs for patients after ACLR. They found that patients who underwent objective-care programs performed better in peak torque values in flexion and extension [21]. B. Sole et al. (2023), in their research, also concluded that rehabilitation should be adapted to every situation and should not be universal [22]. A. Leung et al. (2023) also reported similar thoughts [23]. During the rehabilitation process, rational implications should be made regarding the graft type used, as clinical milestones after surgery differ between them. When QT or BPTB grafts are being used, rehabilitation should include considerations for surgically induced tendinopathy. To promote tissue repair, muscles should be given constant contractions from the early stages. Specific rehabilitation programs should last longer than 2–3 months post-operatively, and the flexion-extension mechanism should be evaluated and treated constantly. Measuring knee flexion and extension torque preoperatively and prescribing a prehabilitation protocol can also help minimize postoperative strength deficits and favor a healthy H/Q ratio to prevent reinjury [1,24].

Patient satisfaction is a key value to determine the success of surgical intervention [25]. Unfortunately, there are not many studies that evaluate it after ACLR, and especially after reconstruction with the quadriceps tendon [1,25]. In our study, patients presented lower scores in the QT group, but only the IKDC score was statistically significantly higher in the HT autograft group. We believe that an outcome of residual quadriceps weakness resulted in unusual H/Q ratio differences between the legs. O. Gorschewsky et al. (2007), in their article, used the Lysholm and IKDC scores (although as functional) to evaluate BPTB and QT with bone block (QTB). They found that patients receiving BPTB autografts were more likely to have a normal score compared with QTB autograft [26]. For patient satisfaction evaluations, unlike us, they created a grading scale and concluded that very good results were more commonly achieved in the BPTB group. However, it is difficult to compare their study and ours because they compared QTB with BPTB, and we compared all-soft-tissue QT with HT. Unfortunately, we did not manage to find studies that purely compare satisfaction between QT and HT.

In a study by Dadoo S et al. (2024), 80% of patients successfully returned to sports at a mean time of 9.7 months, of which 85% of patients returned to the same or a higher level of activity postoperatively [27]. The rate of return to sports (RTS) was similar to previously reported rates of RTS among adolescent athletes after ACLR with QT and other autografts, ranging from 60% to 100% [28,29,30,31]. In addition, the study by Dadoo S et al. (2024) revealed that patients with available pre- and postoperative IKDC scores achieved a significant increase in postoperative IKDC scores (88.5 vs. 37.5; *p* < 0.001) [27]. It presents a strong foundation to support QT autograft for ACLR.

Despite the advantages of our study, such as the homogeneous QT and HT groups and the prospective nature of the design, there are also some limitations, such as the small sample size and a short follow-up interval of only 12 months, rendering long-term conclusions difficult to make. In future studies, a larger sample should be considered. Perhaps different rehabilitation tactics should be applied, keeping in mind that two different reconstruction techniques are used, and the patient follow-up should continue for longer than 12 months due to persistent quadriceps weakness.

## 5. Conclusions

In conclusion, in our study, we found that, after ACLR, the peak torque of flexion is significantly weaker in the HT group, and the peak torque of extension is significantly weaker in the QT group. Quadriceps tendon harvesting is associated with a more favorable H/Q ratio; however, several influencing factors should be considered. Due to the surgically induced weakening of the quadriceps muscle, we obtain a much better and almost ideal H/Q ratio in the QT group. Theoretically, this should significantly reduce the risk of re-rupture, but in practice, it does not reveal the real strength of the hamstrings. When evaluating patient satisfaction, only the IKDC score shows significant results in favor of HT. We believe this is because of residual surgical tendinopathy to the quadriceps muscle. To further clarify which graft is better, we need more and longer studies with larger samples aiming to evaluate the patient function and satisfaction rate after ACLR with QT grafts. Also, the role of different rehabilitation programs for different reconstruction techniques should not be forgotten.

## Figures and Tables

**Table 1:** Sample characteristics between QT and HT autograft groups.

	QT (n = 24)	HT (n = 30)	Test Statistic	*p* Value
Age in years (median (IQR))	15.5 (15–16.75)	15 (14–16)	|Z| = 1.306	0.192
Sex (male/female)	9/15	18/12	χ^2^ = 1.875	0.171
Height in cm (median (IQR))	173.5 (169–181.5)	177.5 (161–180)	|Z| = 0.157	0.875
Weight in kg (median (IQR))	65 (53.5–77.25)	66.5 (62–78)	|Z| = 0.471	0.638
BMI Z-score (median; (IQR))	0.14 (−0.456–0.79)	0.43 (0.21–0.83)	|Z| = 1.255	0.209
Meniscus (repaired/repair not required)	12/12	15/15	χ2 = 0	1

QT: quadriceps tendon autograft, HT: hamstring tendon autograft, |Z|: absolute standardized Mann–Whitney test statistic, χ^2^: Chi-square test statistic.

**Table 2:** Muscle strength outcomes between QT and HT autograft groups.

	QT (n = 24) (Median; IQR)	HT (n = 30) (Median; IQR)	Test Statistic	*p* Value
	6 months post-surgery			
Extensors peak TQ/BW in %	121.45 (92.05–191.4)	156.9 (109.3–260)	|Z| = 2.353	**0.019**
Flexors peak TQ/BW in %	84.35 (49.65–110.56)	75.2 (61.5–98)	|Z| = 0.471	0.638
H/Q Ratio	56.1 (41.93–102.43)	43.75 (39–55.7)	|Z| = 2.353	**0.019**
	12 months post-surgery			
Extensors peak TQ/BW in %	208.55 (136.55–222.2)	250.8 (168–332.1)	|Z| = 3.375	**<0.001**
Flexors peak TQ/BW in %	115.3 (91.98–122.95)	91.25 (80.7–123.4)	|Z| = 1.569	0.117
H/Q Ratio	57.2 (43.98–70.1)	40.9 (37.2–49.7)	|Z| = 4.238	**<0.001**

HT: hamstring tendon; QT: quadriceps tendon; TQ/BW: peak torque to body weight ratio; |Z|: absolute standardized Mann–Whitney test statistic. Highlighted *p* values represent statistical difference.

**Table 3:** Muscle strength outcomes between operated and healthy legs in the QT and HT autograft groups.

	QT (n = 24) (Median; IQR)		Test Statistic	*p* Value
	6 months post-surgery			
	Involved	Uninvolved		
Extensors peak TQ/BW in %	121.45 (92.05–191.4)	175.4 (162.2–256.65)	|Z| = 3.434	**<0.001**
Flexors peak TQ/BW in %	84.35 (49.65–110.56)	84.35 (49.63–126.33)	|Z| = 0.773	0.455
H/Q Ratio	56.1 (41.93–102.43)	49.6 (39.7–56.9)	|Z| = 3.177	**<0.001**
	12 months post-surgery			
	Involved	Uninvolved		
Extensors peak TQ/BW in %	208.55 (136.55–222.2)	252.4 (204.18–290.28)	|Z| = 3.869	**<0.001**
Flexors peak TQ/BW in %	115.3 (91.98–122.95)	116 (89.53–145.05)	|Z| = 2.146	**0.031**
H/Q Ratio	57.2 (43.98–70.1)	50.65 (45.2–55.78)	|Z| = 2.661	**0.006**
	**HT (n = 30)** **(Median; IQR)**			
	6 months post-surgery			
	Involved	Uninvolved		
Extensors peak TQ/BW in %	156.9 (109.3–260)	232.1 (193.1–261.8)	|Z| = 4.478	**<0.001**
Flexors peak TQ/BW in %	75.2 (61.5–98)	102.8 (89.2–126.2)	|Z| = 4.355	**<0.001**
H/Q Ratio	43.75 (39–55.7)	47.2 (43.1−48.4)	|Z| = 1.390	0.165
	12 months post-surgery			
	Involved	Uninvolved		
Extensors peak TQ/BW in %	250.8 (168–332.1)	293 (249.1–322)	|Z| = 3.490	**<0.001**
Flexors peak TQ/BW in %	91.25 (80.7–123.4)	128.95 (97.8–153.6)	|Z| = 4.787	**<0.001**
H/Q Ratio	40.9 (37.2–49.7)	48.4 (44.9–50.2)	|Z| = 3.366	**<0.001**

HT: hamstring tendon; QT: quadriceps tendon; TQ/BW: peak torque to body weight ratio; |Z|: absolute standardized Wilcoxon signed-rank test statistic. Highlighted *p* values represent statistical difference.

**Table 4:** Patient satisfaction results between autograft groups.

	QT (Median; IQR)	HT (Median; IQR)	Test Statistic	*p* Value
IKDC	87.94 (85.64–93.68)	93.13 (88.51–98.85)	|Z| = 2.599	**0.009**
Lysholm	95 (91–100)	98 (95–100)	|Z| = 1.487	0.137
ACL-RSI	70.67 (52.07–88.54)	80.82 (70.83–92.5)	|Z| = 1.570	0.116

QT: quadriceps tendon autograft, HT: hamstring tendon autograft, |Z|: absolute standardized Mann–Whitney test statistic. Highlighted *p* values represent statistical difference.

## Data Availability

The datasets generated and analyzed during the current study are not publicly available due to privacy reasons, but they are available from the corresponding author on reasonable request.

## Abbreviations

ACL	Anterior cruciate ligament
ACLR	Anterior cruciate ligament reconstruction
QT	Quadriceps tendon
HT	Hamstring tendons
IQR	Interquartile range
H/Q	Hamstring-to-quadriceps ratio
TQ/BW	Peak torque to body weight ratio
IKDC	International Knee Documentation Committee score
ACL-RSI	Anterior cruciate ligament return to sport after injury scale
